# Does Microscopic Extrathyroidal Extension Confer a Higher Risk of Recurrence in Patients With Well-Differentiated Thyroid Cancer?

**DOI:** 10.1210/clinem/dgz121

**Published:** 2020-06-29

**Authors:** Furio Pacini

**Affiliations:** Istituto Clinico Humanitas, Humanitas University, Rozzano (Milano), Italy

The tumor-node-metastasis (TNM) American Joint Committee on Cancer (AJCC) staging system for well-differentiated thyroid cancer (WDTC) is the primary staging system for stratification of the risk of death, but not of recurrence. Over time, several changes have been introduced in the different editions of the system, mainly limited to the tumor (pT) classification, highlighting the uncertainty of this as a sole indicator for accurately predicting the risk of death.

The most recent edition (8th) of the AJCC staging system for WDTC has removed microscopic extrathyroidal extension (mETE) from the definition of pT3 disease. T3a and T3b categories were introduced for tumors > 4 cm limited to the thyroid gland and tumors with macroscopic ETE limited to the strap muscles, respectively (1). As a consequence, patients with T3 mETE by the 7th edition staging are redistributed to T1 to T3a, based on tumor size by the 8th edition criteria. These changes reflect evidence that mETE cannot be reliably identified on histopathology, and that when present, its status as an independent prognostic factor in PTC is increasingly under question (2–6). Two retrospective studies have reported that mETE does not affect either the risk of death or the risk of recurrence (2, 6). Nevertheless, the American Thyroid Association (ATA) guidelines for predicting recurrence consider patients with mETE to be at intermediate risk of recurrence (7), suggesting that these patients should be followed and probably treated with more aggressive protocols, including routine or selected use of radioiodine ablation.

A recent retrospective study by Tran et al (8) compared the risk of recurrence of pT3 tumors according to the 7th edition of the AJCC staging system versus the 8th edition. The authors found that pT8 was inferior to pT7 in patients > 55 years of age without macroscopic ETE or distant metastases in whom T classification affects TNM stage. Microscopic ETE was strongly associated with other adverse prognostic factors and reduction of disease-free survival (DFS) in this patient subgroup. The presence of mETE conferred a 2.24-fold increased risk of recurrence for all ages (*P* = 0.009) and a 2.76- fold increased risk in patients aged > 55 years who had no distant metastasis (*P* = 0.002) when adjusted for tumor size. Microscopic ETE was strongly predictive of recurrence on univariate analysis and was an effective surrogate for other adverse risk factors, such as lympho-vascular invasion, multifocality, positive margins, nodal metastasis, and extranodal extension. The authors concluded that their results support the inclusion of mETE in risk stratification models, such as in the previous AJCC TNM editions and the ATA risk of recurrence guidelines (7, 9). A possible criticism of this conclusion is that mETE was not an independent prognostic factor on a full multivariate analysis, which reduced the power of this factor as prognostic index of DFS. The inclusion of a biological parameter among prognostic risk factors based solely on the evidence that it has a close association with multiple other adverse risk factors is not consistent with the concept of evidence-based medicine. As commonly observed in the field of WDTC, we rely mainly on retrospective studies, which often conflict and carry the risk of several, sometimes important, possible biases. As stated by the authors, one bias is that mETE is not reliably identified on histopathology. Apart from speculation, the real point, in my opinion, is whether the inclusion of mETE among risk factors for recurrence would change the management of the disease. According to the ATA risk classification, pT3 (minor ETE) is attributed a risk of recurrence of 3.8%, which falls just at the border between the low-risk and intermediate-risk categories, and in the text it is attributed to the low-to-intermediate risk category, well reflecting the uncertainty of the authors. What are the implications for therapy? The only one that I can envisage is the use of radioactive iodine ablation (RAI), provided that the patient has undergone total thyroidectomy. The ATA guidelines state that in the intermediate category there are “conflicting observational data” that RAI may improve DFS, and the conclusion is that RAI is “generally favored based on risk of recurrent disease. Smaller tumor with mETE may not require RAI.” According to this last sentence, only a minority of the patients in the Tran study (8) would be considered for RAI. Here there is an additional variable: the size of the tumor, which again is a possible bias in retrospective studies. In the era of risk-tailored management, the new paradigm to decide the optimal treatment and follow-up is the ongoing risk stratification based on the response to initial treatment. Using measurement of serum thyroglobulin on l-thyroxin therapy and neck ultrasound some months after initial treatment, we can assess the real risk of recurrence, regardless of the initial risk stratification. An excellent response (undetectable or low serum thyroglobulin and negative neck ultrasound) after total thyroidectomy with or without RAI ablation defines a patient at very low risk of recurrence, not requiring further treatment or strict follow-up. Most patients with mETE will be in this category. In the absence of rigorous prospective studies, this strategy should be the standard of care for patients with minimal ETE and should be adopted.

## Abbreviations

AJCC: American Joint Committee on Cancer
ATA: American Thyroid Association
DFS: disease-free survival
ETE: extrathyroidal extension
mETE: microscopic extrathyroidal extension
RAI: radioactive iodine ablation
TNM: tumor-node-metastasis
WDTC: well-differentiated thyroid cancer

## Additional Information

***Disclosure Summary:*** The author has nothing to disclose.

***Data Availability:*** Data sharing is not applicable to this article as no datasets were generated or analyzed during the current study.
